# Tissue-specific signatures of metabolites and proteins in asparagus roots and exudates

**DOI:** 10.1038/s41438-021-00510-5

**Published:** 2021-04-01

**Authors:** Stefanie Döll, Roxana Djalali Farahani-Kofoet, Rita Zrenner, Andrea Henze, Katja Witzel

**Affiliations:** 1 Leibniz Institute of Plant Biochemistry, Weinberg 3, 06120 Halle/Saale, Germany; 2 Leibniz Institute of Vegetable and Ornamental Crops, Theodor-Echtermeyer-Weg 1, 14979 Großbeeren, Germany; 3 University of Potsdam, Institute of Nutritional Science, Arthur-Scheunert-Allee 114-116, 14558 Nuthetal, Germany; 4 Present Address: German Centre for Integrative Biodiversity Research (iDiv) Halle-Jena-Leipzig, Deutscher Platz 5e, 04103 Leipzig, Germany

**Keywords:** Plant physiology, Plant transporters

## Abstract

Comprehensive untargeted and targeted analysis of root exudate composition has advanced our understanding of rhizosphere processes. However, little is known about exudate spatial distribution and regulation. We studied the specific metabolite signatures of asparagus root exudates, root outer (epidermis and exodermis), and root inner tissues (cortex and vasculature). The greatest differences were found between exudates and root tissues. In total, 263 non-redundant metabolites were identified as significantly differentially abundant between the three root fractions, with the majority being enriched in the root exudate and/or outer tissue and annotated as ‘lipids and lipid-like molecules’ or ‘phenylpropanoids and polyketides’. Spatial distribution was verified for three selected compounds using MALDI-TOF mass spectrometry imaging. Tissue-specific proteome analysis related root tissue-specific metabolite distributions and rhizodeposition with underlying biosynthetic pathways and transport mechanisms. The proteomes of root outer and inner tissues were spatially very distinct, in agreement with the fundamental differences between their functions and structures. According to KEGG pathway analysis, the outer tissue proteome was characterized by a high abundance of proteins related to ‘lipid metabolism’, ‘biosynthesis of other secondary metabolites’ and ‘transport and catabolism’, reflecting its main functions of providing a hydrophobic barrier, secreting secondary metabolites, and mediating water and nutrient uptake. Proteins more abundant in the inner tissue related to ‘transcription’, ‘translation’ and ‘folding, sorting and degradation’, in accord with the high activity of cortical and vasculature cell layers in growth- and development-related processes. In summary, asparagus root fractions accumulate specific metabolites. This expands our knowledge of tissue-specific plant cell function.

## Introduction

Plant roots secrete a wide range of compounds that function in the mobilization of low-availability nutrients from the soil or govern interaction with other organisms in the rhizosphere. Metabolites may be exported to the soil by diffusion along a concentration gradient, by channel proteins, or by ATP- or proton-driven transporters against a concentration gradient^1^. Numerous transporters have been identified which govern the exudation of specific compounds, mostly in the plasma membrane of cells^2^. However, it is largely unknown where these exometabolites are synthesized within the root, the specificity of root exudation with respect to different compound classes, and how the biosynthetic pathways are spatially partitioned within the root. Considerable efforts have been made to profile the array of released compounds^3,4^. Especially secondary metabolites are important molecules with significant impact on the rhizosphere ecosystem, acting as allelochemicals that are exuded to mediate plant growth in the vicinity^5^ or to mobilize nutrients^6^. They also act as signaling molecules that attract or repel microorganisms in the rhizosphere^2,7^. The roles of some groups of secondary metabolites are well described in this context, e. g., flavonoids, strigolactones, or terpenes^8^, while others are less understood. Such specialized metabolites are often accumulated in particular anatomical structures and cell types of the root^9^ but information about the localization of metabolites is lost when homogenized sample material is investigated. It is crucial to track the spatial dynamics of metabolite accumulation to provide insights into tissue and cell type-specific metabolite compartmentalization. However, a systematic assessment of metabolites present in root tissues and the exudate fraction has not yet been attempted in monocot and dicot plant species.

Asparagus (*Asparagus officinalis* L.) is a perennial vegetable consumed worldwide, with a high nutritional value and low-calorie intake. Spears are rich in antioxidants, such as polyphenols, flavonoids, and ascorbic acid as well as amino acids^10–12^, while roots are traditionally used as a medicinal product, mainly due to their accumulation of saponins and fructans^13,14^. Asparagus grows from a root system of fleshy storage roots attached to an underground rhizome. Small feeder roots attached to storage roots absorb nutrients and water and are short-lived, while storage roots continue to grow throughout the plant’s life. Roots exert considerable antimicrobial activities, producing specialized metabolites (steroid-terpenes, alkaloids, flavonoids, among others) that allow shaping their rhizosphere microbiota over the lifespan of an asparagus bed^14^. Asparagus can therefore serve as an excellent model in elucidating long-term rhizodeposition processes.

Here we describe an integrative approach to define tissue-specific resolution in the metabolome and proteome of storage roots. Until now, root metabolite profiling of asparagus focused on selected metabolite classes, such as saponins^15–17^, fructans^18,19^, and flavonoids^20^. Asparagus storage roots consist of an epidermis and a suberized exodermis, the cortex, and the endodermis-surrounded stele^21^. In our approach, roots were dissected into epidermis/exodermis and cortex/vasculature (Fig. S1). The metabolomes of both compartments were compared with that of root exudates. We hypothesized that root tissues have individual metabolite signatures, which differ from those of root exudates. The differentially accumulated metabolite distribution of selected compounds was validated by matrix-assisted laser desorption ionization—mass spectrometry imaging (MALDI-MSI).

Proteins control the biosynthesis of plant metabolites and proteomic techniques have expanded our knowledge about biosynthetic pathways. So far, only transcriptome analyses have been performed in asparagus with the aim to elucidate the biosynthesis of specific compounds^17,22,23^. However, the detection of a particular gene product in a transcript-based experiment does not indicate the presence or absence of the resulting protein product. Further, quantitative differences in the transcript of a particular gene may not necessarily correlate with the corresponding protein abundance or the accumulation of related metabolites. Typical studies measure the protein composition of whole tissue, which leads to an average assessment of the proteome, overlooking cell type-specific dynamics. Only a few cell type-specific proteome studies have aimed at understanding responses to plant development and specific stresses^24^. Therefore, the objective of this study was to investigate the compartmentalization of biosynthetic pathways to enhance our understanding of the intricate regulation of metabolic pathways and networks at the cellular level.

## Results

### Metabolome profiling of asparagus roots and root exudates

Measurements of exudates, outer and inner root tissues by negative ion electrospray ionization (ESI−) led to the detection of 1915, 1437, and 1309 mass/retention time pairs, respectively. ESI+ measurements contained slightly more signals, with 2127, 2413, and 1942 in exudates, outer and inner tissue, respectively. The principal component analysis revealed a distinct separation between the three fractions, for both ionization modes (Fig. S2).

**Fig. 1:**
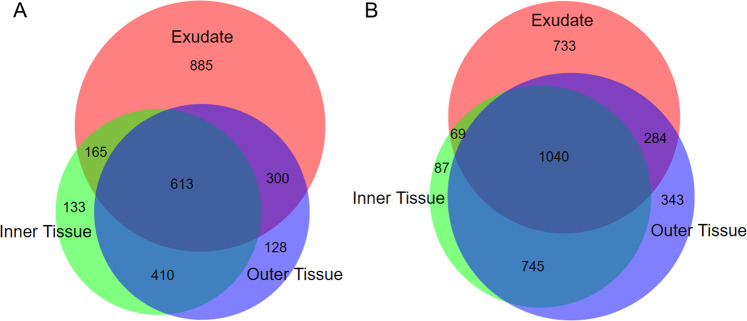
Area-proportional Venn diagrams comparing the number of feature compounds detected in the analyzed root fractions. Metabolite analysis was performed using ESI− (**A**) or ESI+ (**B**)

**Fig. 2:**
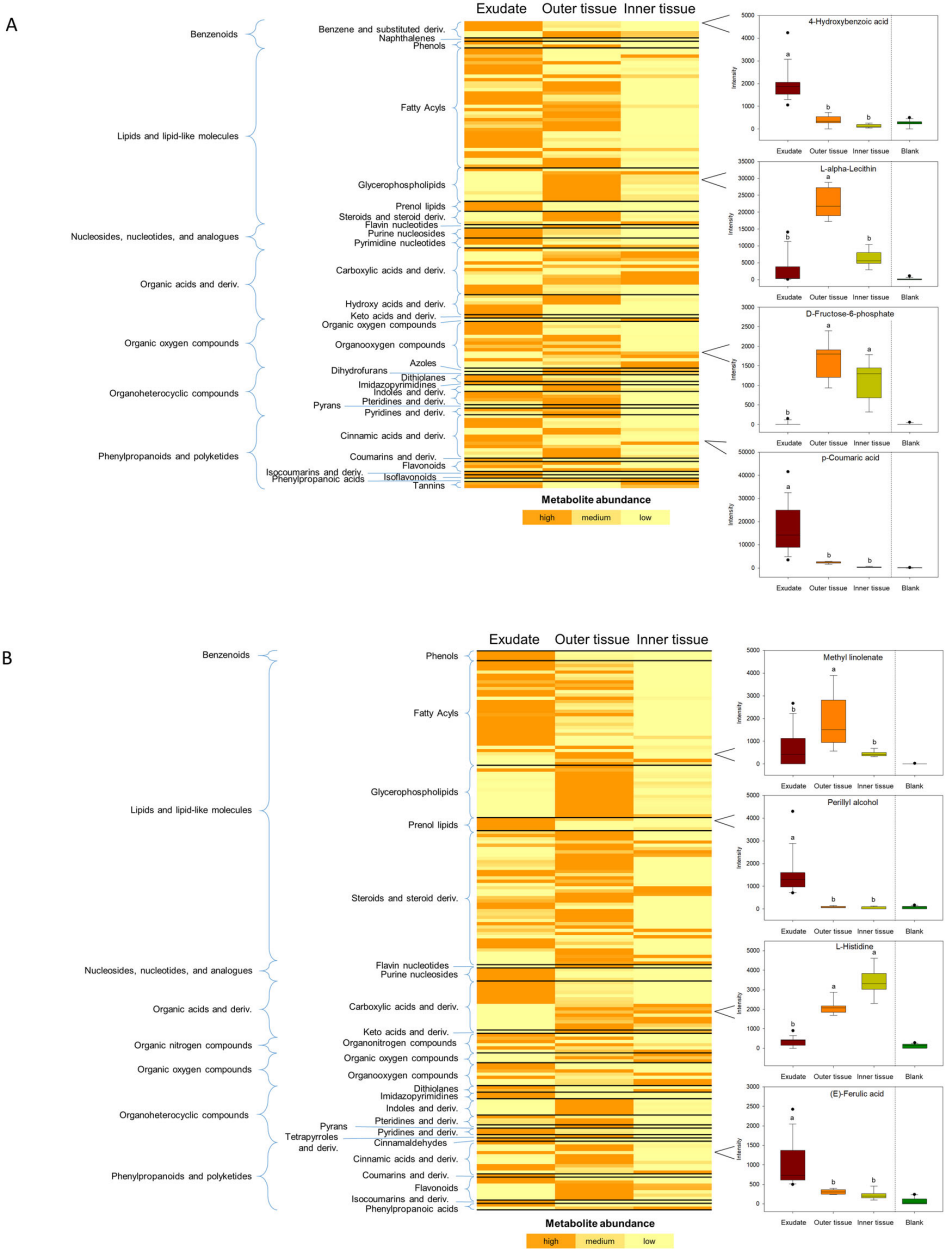
Differentially abundant metabolites (*p* < 0.05, fold change > 2) in the three analyzed root fractions. Compounds were detected by ESI− (**A**) or ESI+ (**B**). Metabolite abundance is presented by a color coding, where orange is the highest abundance, yellow ocher is medium abundance and bright yellow indicates the lowest abundance of the respective metabolite, as mean of all biological and experimental replicates. On the right-hand side, the abundance of selected compounds is shown, based on all replicate measurements (exudate: *n* = 15, outer and inner tissue: each *n* = 9, blank: *n* = 18). The median, 10th, 25th, 75th, and 90th percentiles are plotted as vertical boxes with error bars. Letters indicate significantly different fractions (Kruskal–Wallis One Way ANOVA on Ranks, followed by Dunn’s test for multiple comparisons, *p* < 0.05). Table S1 provides the annotation of the respective compounds, the confidence level of annotation, measured and theoretical masses, retention times, molecular formulas, ion intensities, p values, and compound class annotation up to level 3 subclass. deriv. derivatives

The distribution of compounds between the root fractions was visualized using Venn diagrams. A total of 613 ESI− and 1040 ESI+ signals were common to exudates, outer and inner root tissues (Fig. 1). The largest number of unique features was found for root exudates; more features from outer tissue were common to root exudates as compared to inner tissue. Paired *t*-tests (*p* < 0.05, fold change >2) confirmed the above observation and revealed that metabolite profiles of outer and inner tissue were more similar to each other than to exudates. Table S1 presents those features that were successfully annotated based on their accurate mass and tandem mass spectrum, including their compound class annotation according to KEGG pathway analysis. In total, 263 non-redundant metabolites were found as having significantly different abundances across the three root fractions. Under ESI−, 139 annotated metabolites were significantly changed, with the largest group being ‘lipids and lipid-like molecules’ (38%) and the second largest being ‘phenylpropanoids and polyketides’ (16%, Fig. 2A). Under ESI+, 170 annotated metabolites were significantly changed, with 55% being ‘lipids and lipid-like molecules’ (Fig. 2B). The second-largest group was annotated as ‘phenylpropanoids and polyketides’, accounting for 12% of annotated compounds.

In general, most of the differentially abundant metabolites accumulated either in the outer tissue or in the exudate or both, while only a limited number was enriched in the inner tissue. Prenol lipids and purines were mainly present in root exudates. The identified prenol lipids belonged to mono-, di-, tri- and sesquiterpene groups (including the phytohormones abscisic acid and gibberellin precursor A12) with largely unknown function in the rhizosphere. Purine nucleosides can be transported out of the cells or might be derived from exuded purine nucleotides that have been hydrolyzed by strong extracellular apyrase activity. Glycerophospholipids were enriched exclusively in the outer tissue. These compounds are major structural constituents of cell membranes and root hairs in particular integrate glycerophosphocholines and glycerophosphoethanolamines into their membranes. Steroids and steroid derivatives were also found mainly in outer tissue. Most compounds in this group were annotated as saponins, which represent a plant protective chemical barrier with antimicrobial activity. Fatty acyls and organoheterocyclic compounds were highly abundant in the exudate and epidermis. Most of the annotated fatty acyls were linoleic acids and their derivatives. Free linoleic acid possesses antifungal activity and could have a protective function. Linoleic acid is the most abundant fatty acid in plant membranes and the enrichment reflects the synthesis of new membranes in the epidermis and root hairs. The annotated organoheterocyclic compounds included asparagusic acid, nicotinic acid derivatives, and B vitamins (riboflavin, niacin), which act as regulators for microbial interactions in the rhizosphere, among others. Phenylpropanoids and polyketides were highly abundant in the exudate and outer tissue, annotated mainly as cinnamic acid derivatives which are major components of root waxes that form the lipid barrier on the root surface; *p*-coumaric, ferulic, and caffeic acids are also known allelochemicals and antimicrobials. Different members of other metabolite families were found in all three root fractions, including organic acids and their derivatives, and organic oxygen compounds.

As observed earlier (see Fig. 1), there was an unexpected large degree of difference in metabolite signatures between root outer tissue and root exudate. Some metabolites were detected only in the exudate, probably due to chemical or enzymatically modification of released compounds, and some likely to originate from remaining soil substrate, while others were more than two hundred-fold enriched as compared to outer tissue. The top 30 compounds that accumulated in the exudate are listed in Table S2; there was no specific compound class with enhanced exudation. For most of the enriched metabolites (17), no putative functional role in the rhizosphere has been described. Some are known to have allelopathic (2-hydroxyphenylacetic acid, vanillin) and antimicrobial activities [(E)-1-(4-hydroxy-3-methoxyphenyl)dec-4-en-3-one, (10*E*,12*Z*,15*Z*)-9-oxooctadeca-10,12,15-trienoic acid (9-OxoOTrE), 1-linoleoyl-2-lysophosphatidic acid monomethyl ester 2, caffeic aldehyde, 7-hydroxy-3-(2-hydroxy-propyl)-5-methyl-isochromen-1-one)]. Others act as chemoattractants (thymidine, acetosyringone), function in iron mobilization (fraxetin) or shape the rhizosphere microbiota (hydroxytetradecanoic acid isomer, methyl linoleate, quinic acid).

### Tissue type-specific localization of metabolites in asparagus roots

MALDI-MSI was performed to verify the observed region-specific metabolite accumulation in asparagus roots. Three metabolites were selected for the analysis: riboflavin-5-sulfate and protodioscin, both highly abundant in the outer tissue, and raffinose, which was enriched in the inner tissue. A MALDI-MS method was established using authentic standards of protodioscin and raffinose. For riboflavin-5-sulfate, a riboflavin-5-phosphate standard was used, given the similar molecular masses and the identical MS/MS fragmentation pattern of the riboflavin moiety. α-Cyano-4-hydroxycinnamic acid matrix and negative ionization were used for detection of riboflavin-5-sulfate, with 2,5-dihydroxybenzoic acid matrix and positive ionization for protodioscin and raffinose. The identification of the three substances was based on the molecular mass and MS/MS fragmentation of ions present in methanolic extracts of outer or inner tissue (Fig. S3), since the ion-abundances of riboflavin-5-sulfate and protodioscin were too low for on-tissue MS/MS measurements.

**Fig. 3:**
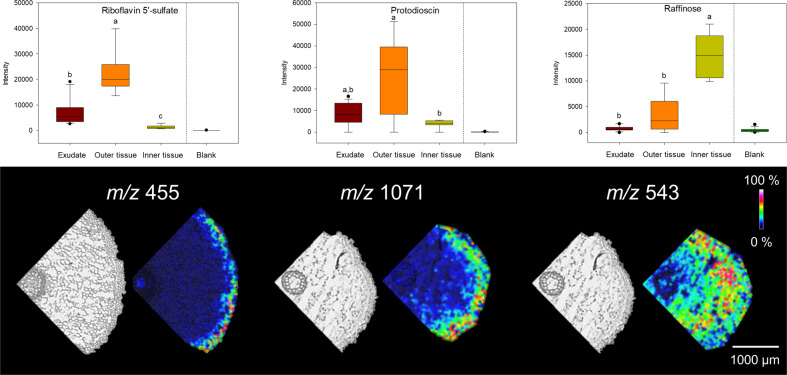
Spatial metabolite distribution in asparagus roots. In the upper row, the abundance of the respective metabolite is given as analyzed by LC-MS. Presented are means of all biological replicate measurements (exudate: *n* = 15, outer and inner tissue: each *n* = 9, blank: *n* = 18) and the median, 10th, 25th, 75th, and 90th percentiles are plotted as vertical boxes with error bars. Letters indicate significantly different fractions (Kruskal–Wallis One Way ANOVA on Ranks, followed by Dunn’s test for multiple comparisons, *p* < 0.05). In the lower row, MALDI-MSI measurements of the respective metabolite are presented. Shown are optical images of root sections (16 µm thickness, left) and MALDI-TOF-MSI images (right) of ions at [M-H]^−^ 455 Da (riboflavin 5’-sulfate), [M + Na]^+^ 1071 Da (protodioscin) and [M + K]^+^ 543 Da (raffinose) at 40 µm resolution

MALDI-MSI confirmed the results of the metabolome profiling (Fig. 3). Riboflavin-5-sulfate was detected exclusively in the outer tissue. The highest abundance of protodioscin was found in the outer tissue, although traces were also detected in the periphery of the cortex. Ion signatures of raffinose were more intense in the cortex as compared to the outer tissue. In order to quantify how strongly those compounds discriminate outer and inner tissue, a receiver operating characteristic (ROC) curve approach was used. The true positive rate of detection was plotted against the false positive rate for each *m/z* value. Area under the curve (AUC) values between 0 and 1 are obtained describing the discriminatory power of an *m/z* value based on its normalized relative abundance. The closer the AUC is to 0 or 1, the higher the discriminatory power of the *m/z* value. AUC values were 0.94 ± 0.03 (±standard deviation, *n* = 6) for riboflavin-5-sulfate, 0.87 ± 0.10 for protodioscin, and 0.72 ± 0.13 for raffinose, indicating that riboflavin-5-sulfate has the highest ability to distinguish between root tissues.

### Spatial distribution of root proteomes

The proteome characteristics of asparagus root outer and inner tissue were investigated using a label-free LC-MS approach. The analysis resulted in 598,054 peptide spectrum matches, indicating 127,241 peptides and 2861 proteins. The principal component analysis revealed a close grouping of technical replicate runs and a clear separation between outer and inner tissue-derived protein samples, explaining 62.7% of the observed variation (Fig. S4). The data set was filtered according to the parameters described in the Materials and Methods section and 1924 identified proteins were subjected to statistical analysis. A total of 104 proteins were found exclusively in outer tissue samples, 76 proteins were found only in the inner tissue, and 405 proteins were differentially abundant (*p* < 0.05, Benjamini-Hochberg corrected for false-discovery rate, Table S3).

**Fig. 4:**
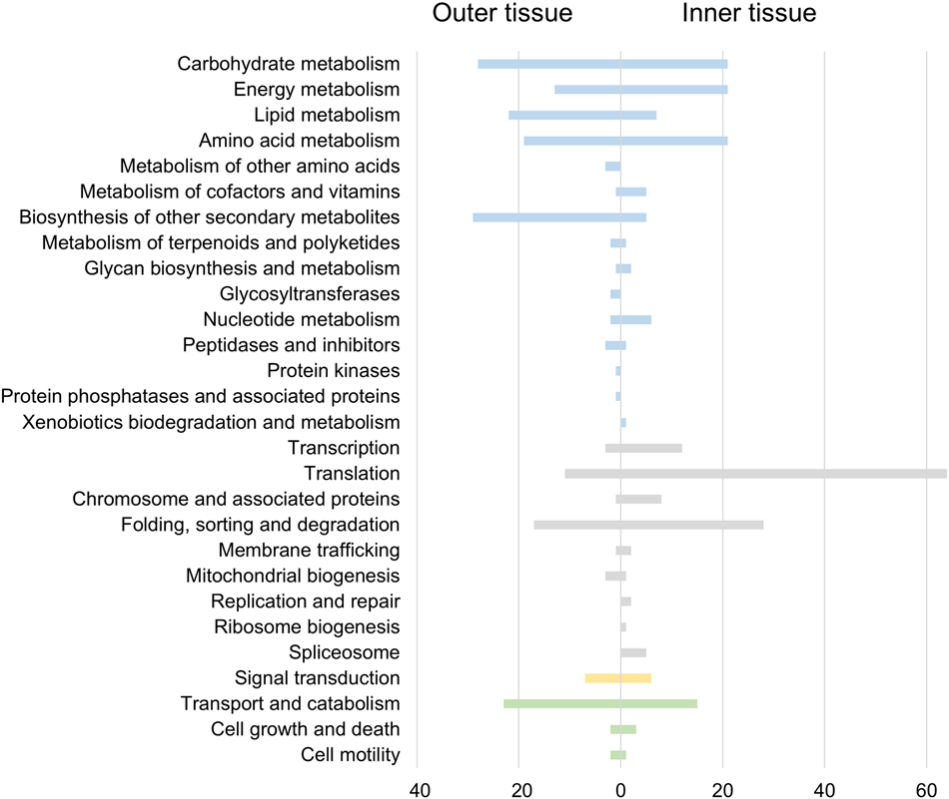
Functional annotation of differentially abundant proteins present in asparagus root tissues. The number of proteins matched to each category is given. Bars are color-coded for major KEGG pathways: metabolism (blue), genetic information processing (gray), environmental information processing (yellow), and cellular processes (green)

In order to characterize individual protein functions, KEGG orthology assignments were performed, permitting annotation of 74.5% of all differentially abundant proteins (Fig. 4, Table S3). Proteins from both tissues were included in the categories ‘carbohydrate metabolism’, ‘energy metabolism’, and ‘amino acid metabolism’, which describe broad and basic metabolic functions. However, differential abundance could be due to the expression of tissue-specific isoforms (e.g., of cysteine synthase, glutamine synthetase, malate dehydrogenase, sucrose synthase), but also due to the enhancement of different cellular processes. For instance, proteins involved in sucrose synthesis (sucrose-phosphate synthase, sucrose-phosphatase) had a higher abundance in the inner tissue, while proteins involved in cell wall-related carbohydrate metabolism (alpha-galactosidase, trifunctional UDP-glucose 4,6-dehydratase/UDP-4-keto-6-deoxy-D-glucose 3,5-epimerase/UDP-4-keto-L-rhamnose-reductase) were more abundant in the outer tissue. The categories that were specifically enriched for outer tissue proteins were ‘lipid metabolism’, ‘biosynthesis of other secondary metabolites’ and ‘transport and catabolism’, reflecting its main function in the formation of a hydrophobic barrier, secretion of secondary metabolites into the rhizosphere, and water and nutrient uptake. Major pathways for inner tissue-derived proteins included ‘transcription’, ‘translation’ and ‘folding, sorting and degradation’, indicating that the cortical and vascular cell layers investigated in our study are highly active in growth- and development-related processes.

To identify proteins that may be involved in root exudation or other processes at the plant-soil interface, the dataset of proteins found exclusively or with higher abundance in the outer tissue was searched for proteins that are located at the plasma membrane or directed to the apoplast. Software tools for predicting the subcellular localization of proteins (CELLO2GO, LOCTree3, TargetP 2.0, WoLF PSORT) yielded contrasting results. Hence, the asparagus protein sequences were blasted against the UniProtKB/SwissProt database, and the putative subcellular localization and putative function were extracted manually based on experimental data of heterologous proteins, if available. Thirty proteins were found that are putatively localized to the plasma membrane, and ten proteins to the apoplast (Table 1). Most prominent were proteins involved in vesicle trafficking and endocytosis, indicating that vesicle transport might be an important mechanism of exudation in asparagus; endosomal interactions also contribute to tip growth of root hairs. Further, proteins related to lipid metabolism, cell wall metabolism, and signaling/stress response were found, which relate to the primary function of the root epidermis as a barrier and boundary between the plant and its environment. Our analysis identified two ATP-binding cassette (ABC) transporter G family proteins (gi:1150689455, gi:1150740767) as highly abundant in the epidermis. The initial proteome dataset contained two additional ABC transporter G proteins that were expressed exclusively in the epidermis (gi:1150748405, gi:1150679400), however, these proteins were each identified by a single peptide and thus did not meet our quality threshold.

**Table 1:** Identification of proteins that are putatively localized to the plasma membrane or the apoplast and that are significantly higher abundant or exclusively expressed (+) in the root outer tissue (*t*-test; *p* < 0.05)

Accession	Description	Putative localization	Putative function	Abundance ratio: outer tissue/inner tissue
gi:1150718353	CASP-like protein 1B1	Plasma membrane	Casparian strip membrane protein	+
gi:1150692480	Dynamin-related protein 1C	Plasma membrane	Cell integrity	+
gi:1150695926	Plasmodesmata callose-binding protein 3	Plasma membrane	Cell-to-cell trafficking	+
gi:1150679111	AP-2 complex subunit alpha-2	Plasma membrane	Clathrin-dependent endocytosis	2.68
gi:1150700055	AP-2 complex subunit sigma	Plasma membrane	Clathrin-dependent endocytosis	2.04
gi:1150700227	O-acyltransferase WSD1	Plasma membrane	Cuticular wax biosynthesis	+
gi:1150707230	vacuolar protein sorting-associated protein 32 homolog 2	Plasma membrane	Endosomal sorting	+
gi:1150735967	Non-specific lipid-transfer protein 8	Plasma membrane	Lipid transfer	+
gi:1150688079	YLS3-like protein	Plasma membrane	Lipid transfer	132.33
gi:1150692863	Cellulose synthase interactive 1-like protein	Plasma membrane	Microtubule binding	+
gi:1150676308	Uncharacterized protein LOC109847110	Plasma membrane	Protein folding	3.90
gi:1150743591	Nicalin	Plasma membrane	Protein processing	2.26
gi:1150725132	LRR receptor-like serine/threonine-protein kinase	Plasma membrane	Signaling	+
gi:1150673097	LYK5-like protein	Plasma membrane	Signaling	+
gi:1150709933	PTI1-like tyrosine-protein kinase	Plasma membrane	Signaling	20.05
gi:1150669161	Uncharacterized protein LOC109827341	Plasma membrane	Signaling	1.72
gi:1150708519	ADP-ribosylation factor 1	Plasma membrane	Vesicle trafficking	7.97
gi:1150704764	Patellin-3	Plasma membrane	Vesicle trafficking	3.02
gi:1150677177	Ras-related protein Rab7	Plasma membrane	Vesicle trafficking	+
gi:1150678524	Ras-related protein RABB1c isoform X1	Plasma membrane	Vesicle trafficking	2.08
gi:1150746456	Ras-related protein RABC1 isoform X1	Plasma membrane	Vesicle trafficking	1.73
gi:1150702568	Ras-related protein RABD1	Plasma membrane	Vesicle trafficking	+
gi:1150682822	Ras-related protein RABE1c	Plasma membrane	Vesicle trafficking	1.98
gi:1150732581	Vacuolar-sorting receptor 1	Plasma membrane	Vesicle trafficking	8.80
gi:1150733323	Vesicle-associated protein 1-1	Plasma membrane	Vesicle trafficking	+
gi:1150689455	ABC transporter G family member 11	Plasma membrane	Unknown	+
gi:1150740767	ABC transporter G family member 39	Plasma membrane	Unknown	26.03
gi:1150695579	Calcium-transporting ATPase 1	Plasma membrane	Unknown	5.92
gi:1150716597	Plastidic glucose transporter 4	Plasma membrane	Unknown	4.68
gi:1150716064	Uncharacterized protein LOC109844061	Plasma membrane	Unknown	6.58
gi:1150704156	Polygalacturonase-like protein	Extracellular	Cell wall	+
gi:1150687475	Probable polygalacturonase	Extracellular	Cell wall	32.90
gi:1150688756	GDSL esterase/lipase	Extracellular	Lipid metabolism	+
gi:1150700397	GDSL esterase/lipase	Extracellular	Lipid metabolism	79.00
gi:1150728688	Purple acid phosphatase 2	Extracellular	Metal binding	+
gi:1150713849	Subtilisin-like protease SBT1.2	Extracellular	Peptidase	2.47
gi:1150714274	Subtilisin-like protease SBT1.2	Extracellular	Peptidase	+
gi:1150709859	Uncharacterized protein LOC109841113	Extracellular	Stress response	+
gi:1150669453	Putative germin-like protein 2-1	Extracellular	Stress response	+
gi:1150694897	Uclacyanin 1	Extracellular	Unknown	+

### Spatial distribution of biosynthetic pathways

**Fig. 5:**
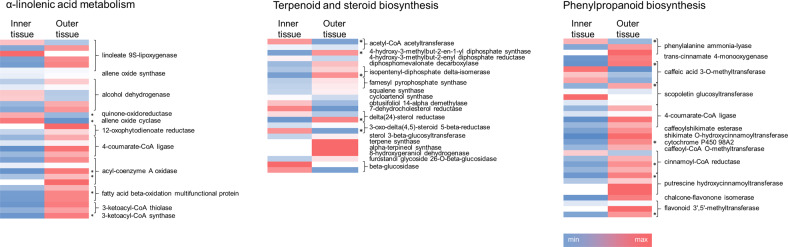
Heatmaps depicting proteins that were identified by the proteome analysis as involved in the biosynthetic pathways of α-linolenic acid, terpenoid and steroid, and phenylpropanoid biosynthesis. Colors represent the normalized protein expression ranging from minimum (dark blue) to maximum (dark red). Missing colors indicate the absence of the protein in the respective tissue. Asterisks indicate significantly different protein abundances (*t*-test, *p* < 0.05). Further information related to protein accession numbers, abundance ratio, and significance testing is provided in Supplementary Table S5

Several metabolite classes were identified that showed a tissue type-specific accumulation (Fig. 2). To gain more insight into these distribution patterns, proteins related to their underlying biosynthetic pathways were examined, using the asparagus KEGG pathways for α-linolenic acid metabolism, steroid biosynthesis, and phenylpropanoid biosynthesis (Fig. 5, Fig. S4). α-Linolenic acid is a polyunsaturated fatty acid, structural component of storage and membrane lipids, and a precursor of the signaling molecule jasmonic acid. A number of proteins involved in earlier metabolic steps were exclusively expressed or had significantly higher abundance in the inner tissue, including a linoleate 9S-lipoxygenase isoform (gi:1150749578), quinone-oxidoreductase (gi:1150698529), and allene oxide cyclase (gi:1150676538). Downstream proteins related to (15Z)-12-oxophyto-10,15-dienoate metabolism were more highly abundant in the outer tissue; a 12-oxophytodienoate reductase isoform (gi:1150734677) and one acyl-coenzyme A oxidase isoform (gi:1150714278) were exclusively expressed there.

Since the metabolome analysis identified a large number of steroidal saponins, the terpenoid and steroid biosynthetic pathway was examined. Three proteins involved in monoterpenoid metabolism were expressed only in the outer tissue (terpene synthase, gi:1150739266; alpha-terpineol synthase, gi:1150721196; 8-hydroxygeraniol dehydrogenase, gi:1150697747). No clear tissue-specific localization was detected for saponin biosynthesis: one delta(24)-sterol reductase isoform (gi:1150724297) had significantly higher abundance in the outer tissue, while a 3-oxo-delta(4,5)-steroid 5-beta-reductase isoform (gi:1150723526) had higher abundance in the inner tissue.

Phenylpropanoid metabolism generates a vast array of secondary metabolites. One scopoletin glycosyltransferase (gi:1150677396) was exclusively expressed in the inner tissue. Proteins involved in the synthesis of monolignols were more abundant in the outer tissue, including cinnamoyl-CoA reductase (gi:1150709547) and caffeic acid 3-O-methyltransferase (gi:1150739025). Proteins involved in the generation of hydroxycinnamic acid amides, putrescine hydroxycinnamoyltransferase isoforms (gi:1150715986, gi:1150711748, and gi:1150715984), had higher abundance or were exclusively expressed in the outer tissue.

We further investigated the localization of enzymes involved in the biosynthesis and metabolism of different types of specialized metabolites (Table S5). Glycosyltransferases govern the transfer of a glycosyl moiety to a substrate compound, which can be phenylpropanoids, flavonoids, hormones, or xenobiotics. Thirteen glycosyltransferases have been identified in asparagus roots by the proteome approach. For most, their specific substrate is unknown but deduced based on sequence similarity searches against UniProtKB. Two proteins were tissue-specific: UDP-glycosyltransferase 92A1 (gi:1150750624) with unknown substrate specificity being expressed in the outer tissue and scopoletin glucosyltransferase (gi:1150677396) found only in the inner tissue. Six proteins were significantly differentially expressed between the tissues, all of them more abundant in the outer one. Notably, the increased occurrence of UDP-glycosyltransferases in the outer tissue was accompanied by a higher abundance of their co-substrate UDP-glucose. Cytochrome P450 monooxygenases mediate multiple oxidative processes, especially in the biosynthesis of specialized metabolites. Twelve cytochrome P450 enzymes were found in this analysis. P450 71A1 (gi:1150681655), involved in cyanogenic glycoside biosynthesis, was exclusive to the outer tissue. Three enzymes had significantly higher expression in this tissue, two involved in phenylpropanoid metabolism and one in fatty acid biosynthesis (gi:1150714305, gi:1150734984, gi:1150748158). P450 90B1 (gi:1150690507), functioning in brassinosteroid metabolism, was significantly highly expressed in the inner tissue. Glutathione-S-transferases (GST) and glutathione conjugation have multiple functions in plants, including detoxification processes and oxidative stress alleviation. However, they are also essential for the vacuolar accumulation of phenylpropanoids as well as for transport processes via ABC transporters. Sixteen GSTs were identified in asparagus roots, but the substrate specificity for most of them is unknown. Four GST isoforms were identified exclusively in the outer tissue and four other isoforms were significantly upregulated there. Four GST isoforms had higher abundance in the inner tissue, with two of them functioning in scavenging reactive oxygen species via ascorbate (gi:1150727243, gi:1150682857). Overall, from the 41 proteins with putative activity as UDP-glycosyltransferases, cytochrome P450 enzymes, or GSTs, 31 had higher abundance in the outer tissue, reflecting the enhanced synthesis, storage, and transport of specialized metabolites.

## Discussion

This study demonstrates that the analysis of specific root fractions provides valuable insights into cellular function. Metabolites in plant roots exert a variety of functions, such as fueling primary metabolism and root growth, rhizosphere communication, and plant defense. By combining metabolomics and proteomics, we were able to dissect specific metabolic profiles in the three analyzed fractions, and relate those profiles with protein abundances of spatially resolved biosynthetic pathways.

Most differentially abundant compounds found in our study were annotated as ‘lipids and lipid-like molecules’ that accumulate mainly in the outer tissue and the exudate of asparagus roots. Plant lipid metabolism generates compounds with functions in surface protection, intra- and extracellular signaling, membrane organization, and environmental adaptation^25,26^. α-Linolenic acid metabolism is an essential pathway in this regard. Underlying biosynthetic proteins were also more abundant in the outer tissue. Together with the increased abundance of proteins involved in vesicle trafficking, this reinforces the role of these cells in synthesizing and secreting lipids and their derivatives.

Purines and pyrimidines were predominantly found in exudates of asparagus roots. It is known that nucleosides and nucleobases can pass plant membranes via several transport proteins^27^, and plant roots can take up and metabolize nucleosides for degradation, or utilize them in more efficient salvaging processes^28^. However, the extracellular nucleotide ATP has been identified as a plant-surface signaling molecule, with functions in stress and wounding responses of roots^29–31^.

The limited lifespan of an asparagus bed and the problematic replanting are in part associated with the accumulation of pathogenic soil-borne microorganisms, such as *Fusarium* species, and also with the root exudation of autotoxic compounds, including *trans*-cinnamic acid^32^ and caffeic acid^33^. Besides caffeic acid and its derivatives, our study identified several other cinnamic acid derivatives with allelochemical properties accumulated in root exudates (p-coumaric acid, ferulic acid). This could indicate the presence of yet unconsidered autotoxic metabolites relevant for future crop improvement. In general, most differentially abundant compounds belonging to the family of ‘phenylpropanoids and polyketides’ accumulated in the outer tissue and exudate; hydroxycinnamic acids integrate especially into root surface lipids^34^. Concomitant with the presence of metabolites, proteins involved in their biosynthesis were also found more highly abundant or exclusively expressed in the outer tissue, compared to the inner tissue. In particular, a number of putative UDP-glycosyltransferases, cytochrome P450s, and glutathione-S-transferases, catalyzing the final biosynthetic steps of a wide range of compound classes, were outer tissue-specific, indicating a metabolic flow of intermediates from the cortex to the epidermis. Within the cell, secondary metabolites are stored in vacuoles to avoid self-toxification and unspecific compound modifications. Transport to vacuoles and to the apoplast for rhizodeposition occur either in a Golgi-dependent way via vesicle trafficking^35^ or in a Golgi-independent way by specific transporters^36^. Both distribution systems are present in asparagus root outer tissue but their specific roles have not yet been determined.

Saponins usually accumulate in underground tissues of plants and have also been found in root exudates^37^. Saponins are part of the constitutive defense system acting as deterrents, toxins, and digestibility inhibitors^38^. More recently, their role in plant development, especially root growth, root hair morphology, root cap, and root epidermis formation has been described^37^. Metabolite analysis resulted in the annotation as saponins of 42 compounds in ESI+ mode and four compounds in ESI− mode. Most saponins, including protodioscin, were significantly enriched in the outer tissue, while other saponins were more abundant in the exudate or inner tissue. The spatial localization of protodioscin was validated by MALDI-MSI. Protodioscin has multiple medicinal properties^39^. In *Asparagus cochinchinensis* and *Asparagus racemosus*, protodioscin accumulated in all root tissues, but was highest in the epidermis of dried storage roots^16^. A similar epidermis specificity was shown for saponins in *Panax* roots^40^ and avenacins in oat^41^. Despite the clear spatial separation of the metabolite in root tissues, the same degree of separation was not observed for the biosynthetic pathway, as investigated by proteome analysis. Primary enzymes for protodioscin synthesis are cycloartenol synthase (gi: 1150672824) and obtusifoliol 14-alpha demethylase (gi: 1150669044), both having higher abundance in the inner tissue. Neither glucosylation through sterol 3-beta-glucosyltransferase UGT80A2 (gi:1150698245) nor deglucosylation via a furostanol glycoside 26-O-beta-glucosidase (gi: 1150670467) demonstrated tissue specificity, indicating that steroidal saponin synthesis occurs in both tissues and metabolites might be directed to the outer tissue by vesicle transport. Uncompleted biosynthesis of triterpenoid avenacins in oat disrupts membrane trafficking and causes reduced root growth and root hair deficient phenotypes^41^, but comparable insights into steroidal saponin sequestration are lacking.

Numerous studies have investigated proteome responses of entire and developing plant roots towards biotic or abiotic stresses but tissue and cell type-specific investigations are scarce. Root hair cells have become a model to study single-cell proteomes due to the relative simplicity of their preparation and separation from the epidermis^42–44^. In contrast to this and probably due to the relatively low amount of cells required, transcriptome analyses of specific root tissue and cell types have been applied to a greater extent, shedding light on cell differentiation and functioning^45^. We demonstrate in our study that tissue type-specific proteome analyses are particularly useful for studying the molecular mechanisms of processes, which have varied effects on different layers of root cells, similar to the studies on microdissected root tissues of tomato^46^. However, taking into account the broad range of compound classes released from asparagus roots, the number of potential plasma membrane transporters identified in our study was relatively low. Subcellular proteome analysis of enriched plasma membranes from root epidermis would reveal numerous new candidates for the rhizodeposition process^47^ improving knowledge on import and export activities at the plasma membrane. Further, the present proteome study does not differentiate between the epidermis and root hairs, the latter representing tubular extensions of epidermal cells that largely account for import and export processes in the root. Thus, epidermis cells and root hairs should be analyzed separately, as has been demonstrated for *Arabidopsis* roots^48^.

## Materials and methods

### Plant material and growth conditions

Plants were grown as previously described^49^. Briefly, seeds of *Asparagus officinalis* L. ‘Backlim’ were sown in trays containing a 1:2 mixture of sand and standardized plant growth substrate (Fruhstorfer Erde type P, Germany) and cultivated at 25 °C in the dark until the first stem developed. Plantlets were then exposed to 25/20 °C, 75/85% relative humidity and a 12/12 h day-night-cycle with a light intensity of ca 400 µMol m^-2^ s^-1^ and watered as required. After the development of the third stem, single plants were transferred to pots containing a 1:1 mixture of sand and standardized plant growth substrate and cultivated at 23/18 °C and 75/85% relative humidity with a 16/8 h photoperiod (ca 400 µMol m^−2^ s^−1^). The experiment was performed in triplicate.

### Collection of root exudates and separation of root tissues

Root exudates were collected from five plants per experiment (*n* = 15) using a protocol modified from Xu et al.^50^. The cultivation substrate was carefully removed and the plants transferred to glass beakers filled with distilled water. After 1 h, plants were transferred to fresh glass beakers filled with double-distilled water (ca 1 L) and exudates were collected for 3 h. During this time, the water in the beakers was aerated. Exudates were filtered through a 0.22 µm mixed cellulose ester membrane (Carl Roth GmbH, Germany) to remove cellular debris and external microorganisms. Exudates were freeze-dried and subjected to metabolite analysis. After the collection of exudates, the roots were harvested and weighed fresh to determine the exudate-root biomass ratio. A control (‘blank’) was carried out without roots.

For the analysis of metabolites and proteins in root epidermis/exodermis (outer tissue) and root cortex/vasculature (inner tissue), tissues were separated using forceps. The quality of preparation was assessed visually (Fig. S1) and microscopically. For metabolite analysis, three plants per experiment were harvested and analyzed (*n* = 9). For proteome analysis, three plants per experiment were harvested and the material was pooled to give one sample per experiment (*n* = 3).

### Metabolite analysis of root exudates and root tissues

The freeze-dried exudates were suspended in 4 mL methanol (LCMS grade, Chromasolv®, Riedel-de Haën, Germany, 80% v/v) and concentrated. The equivalent of 60 g roots was then taken up in 500 µL of 80% methanol (v/v) containing 100 µM ribitol (Biochemica, Germany), with 5 µM kinetin (Roth, Germany), 5 µM biochanin A (Sigma-Aldrich, Germany), and 5 µM IAA-valine (Sigma-Aldrich) as internal standards.

For root tissues, between 50 and 150 mg were blended with 500 µL 80% methanol (v/v) in a pre-cooled homogenizer for 2 × 45 s at 6500 Hz. The debris was sedimented in a tabletop centrifuge at maximum speed and room temperature for 15 min. The pellet was re-extracted with another 500 µL methanol (80% v/v) and the supernatants combined. The sample was taken to dryness at 30 °C in a concentrator, redissolved in 500 µL methanol (80% v/v with internal standards) per 100 mg starting material using 5 min in an ultrasonic bath, 15 min shaking and another 5 min in the ultrasonic bath. All samples for LC-MS analysis were suspended in HPLC mobile phase A (water (Chromasolv LC-MS ultra, Honeywell/Riedel-de Haën) with 0.1% v/v formic acid; LCMS HiPerSolv CHROMANORM, VWR, Germany), 80–20 sample-solvent), incubated overnight at −20 °C, centrifuged for 10 min at maximum speed and filled into LC vials.

LC-ESI-Q-TOF-MS measurements were based on Böttcher et al.^51^ with several modifications: chromatographic separations were performed at 40 °C on an Acquity UPLC system (Waters) equipped with an HSS T3 column (100 × 1 mm, 1.8 µm; Waters, Germany) applying the following binary gradient at a flow rate of 150 µL min^−1^: 0 to 1 min, isocratic 95% A (as above), 5% B [acetonitrile:formic acid: 99.9:0.1 (v/v)]; 1–18 min, linear from 5 to 95% B; 18 to 20 min, isocratic 95 % B. The injection volume was 3.1 μL (full loop injection). Eluted compounds were detected from *m/z* 90 to 1600 at a spectrum rate of 3 Hz, (line spectra only) using a MicroTOF-Q II hybrid Q-TOF-MS (Bruker Daltonik, Germany). The instrument settings for ESI− were: nebulizer gas, nitrogen, 1.6 bar; dry gas, nitrogen, 6 L min^−1^, 190 °C; capillary voltage, 4000 V; end plate offset, 500 V; funnel 1 radio frequency (RF), 200 Volts peak-to-peak (Vpp); funnel 2 RF, 250 Vpp; in-source collision-induced dissociation (CID) energy, 0 eV; hexapole RF, 120 Vpp; quadrupole ion energy, 3 eV; quadrupole low mass, 100 *m/z*; collision gas, nitrogen; collision energy, 8 eV; prepulse storage, 7 µs. Stepping: on; basic mode; collision cell RF, from 120 Vpp to 350 Vpp; transfer time, from 55 to 90 µs; 70%/30%, collision energy for MSMS, always 100%. For ESI+ the settings were the same, except: capillary voltage, 4500 V; funnel 2 RF, 220 Vpp; quadrupole ion energy, 4 eV; collision energy, 10 eV. Stepping: collision cell RF, from 160 Vpp to 350 Vpp; transfer time, from 60 to 90 µs, collision energy for MSMS, always 80%. Calibration of the *m/z* scale was performed for individual raw data files on sodium formate cluster ions.

For the acquisition of CID (collision-induced dissociation) mass spectra, the same parameters as above were used with additional settings for data-dependent acquisition (AutoMSMS): Mode: CID, intensity threshold 600, number of precursors, 3; precursor background subtraction on, active exclusion on after 3 spectra, release after 1 min, smart exclusion, on, 5x; isolation and fragmentation settings, size and charge dependent, width 3–15 *m/z*, collision energy 10–70 eV, charge states included: 1z, 2z, 3z.

LC-MS and MS/MS data were processed with MetaboScape 4.0 (Bruker Daltonik) using Bruker’s T-ReX 3D algorithm with the following settings: intensity threshold 1500 counts, minimum peak length 7 spectra, feature signal = intensity, mass recalibration auto-detect. Recursive feature extraction: minimum peak length (recursive) 3 spectra, minimum number of features for recursive extraction 6 of 213. Bucket filter: Presence of features in minimum number of analyses 6 of 213.

Annotation of compounds was based on (1) an in-house library of analytical standards and known plant metabolites according to mass, retention time and spectrum; (2) known metabolites from asparagus^12,16^ and the KNApSAcK database^52^, considering mass and spectral similarity for compound class; (3) via spectral similarity to the NIST17 and WEIZMASS databases^53^, Sumner Spectral library (Bruker Daltonik), MoNA (https://mona.fiehnlab.ucdavis.edu/), GNPS (https://gnps.ucsd.edu/), ReSpect^54^ and an in-house database via the spectral library search function of MetaboScape; (4) via similarity towards already annotated compounds from the data set^55^.

PCAs and Student’s *t*-tests were created or calculated with MetaboScape 4.0 (Bruker Daltonik). For *t*-tests, zeros were replaced with the smallest intensity value (20) in the data set. Venn diagrams were created using only compounds that were detected in two of the three experiments replicate samples from the specific root fractions^56^. Box plots and non-parametric tests shown within were created with Sigma Plot 14.0 (Systat Software, Germany).

Metabolome raw data has been deposited at Metabolights (https://www.ebi.ac.uk/metabolights/index) under the dataset ID MTBLS2116.

### MALDI mass spectrometry imaging of root cross sections

Roots from two plants per experiment were harvested for MALDI mass spectrometry analysis (*n* = 6), snap frozen in liquid nitrogen, and stored at −80 °C for further analysis. At least two tissue sections per plant were analysed in MALDI-MSI.

MALDI-MSI was performed as described earlier^57^. In brief, intact roots were transferred to a cryostat (CM3050S, Leica, Germany) with the chamber cooled to −20 °C and the sample holder to −18 °C, and cut at a thickness of 16 µm. These sections were immediately thaw mounted onto a conducting indium tin oxide-coated glass slide (ITO slide, Bruker Daltonik) and held in a desiccator for 15 min. The matrix was *α*-cyano-4-hydroxycinnamic acid (CHCA, Bruker Daltonik) diluted to 7 g L^−1^ in 50% acetonitrile/0.2% trifluoroacetic acid (Sigma-Aldrich), or 2,5-dihydroxybenzoic acid (DHB, Sigma-Aldrich) diluted to 30 g L^−1^ in 50% methanol/0.2% trifluoroacetic acid (Sigma-Aldrich) and was applied to the slide surface by an ImagePrep device (Bruker Daltonik) using the pre-set method for the respective matrix.

MSI experiments were performed using an ultrafleXtreme MALDI-TOF instrument (Bruker Daltonik) run in negative ionization for CHCA matrix or positive ionization for DHB matrix. The laser raster was set to 40 µm and the *m/z* range was 200–1200. For measurements performed in negative mode, the method was calibrated with a mix of authentic standards (1 mM each) of 2-propenyl glucosinolate, [M-H]^−^ 358.05 Da (Carl Roth GmbH & Co. KG, Germany), 3-butenyl glucosinolate, [M-H]^−^ 372.05 Da (Phytolab GmbH & Co. KG, Germany), 4-(methylsulfanyl)butyl glucosinolate, [M-H]^−^ 420.08 Da (Phytolab GmbH & Co. KG), 4-(methylsulfinyl)butyl glucosinolate, [M-H]^−^ 435.05 Da (Phytolab GmbH & Co. KG), rutin, [M-H]^−^ 609.14 Da (Sigma-Aldrich). For measurements performed in positive mode, the method was calibrated with a mix of Peptide Calibration Standard II, Bruker Daltonik Germany, and a polyethylene glycol mixture (1:1 PEG 200 and 600 (Sigma-Aldrich), diluted 1:300 in 30% v/v acetonitrile and 0.1% w/v trifluoroacetic acid). Measurements were performed by flexControl v3.4 software (Bruker Daltonik) and flexImaging v4.0 software (Bruker Daltonik). Total Ion Count (TIC) normalization was applied for MSI data in each pixel.

ROC curve analysis was performed on tissue sections from two plants per replicate (*n* = 6) using SCiLS Lab software (version 2019c Pro, Bruker Daltonik). Tandem mass spectrometry measurements (Lift mode, ultrafleXtreme MALDI-TOF instrument) of selected MSI *m/z* were performed on methanolic extracts of outer and inner tissue and compared with a standard (protodioscin, riboflavin-5-phosphate, raffinose; Sigma-Aldrich).

### Protein extraction and label-free protein quantification of root tissues

Proteins in root epidermis and root tissue were extracted using RapiGest SF (Waters), then reduced and digested following the method of Kaspar et al.^58^. Desalting of peptides was done using Peptide Desalting Spin Columns (Pierce, Thermo Scientific, United States) following the manufacturer’s instructions. Peptides were re-suspended in 2% acetonitrile/0.1% trifluoroacetic acid to a concentration of 100 ng µL^−1^ and 6 µL of protein digest were analyzed using nanoflow liquid chromatography on a Dionex UltiMate 3000 system (Thermo Scientific) coupled to a Q Exactive Plus mass spectrometer (Thermo Scientific) as described previously^59^, with the following modifications. Peptides were separated using an Acclaim PepMap 100 C18 analytical column (75 µm × 25 cm, 2 µm, 100 Å, Thermo Scientific) and eluted via a 100 min gradient from 2 to 44% solvent B (80% acetonitrile). Each sample was measured in triplicate. The raw files were processed using Proteome Discoverer 2.4 and Sequest HT engine (Thermo Scientific), searching the NCBI *A. officinalis* Annotation Release 100 (as released on 1 March 2017). Precursor ion mass tolerance was set to 10 ppm and fragment ion mass tolerance was set to 0.02 Da. False discovery rate (FDR) target values for the decoy database search of peptides and proteins were set to 0.01 (strict level for highly confident identifications). Protein abundance quantification was done using the Top N average method, (*N* = 3). Differential protein expression was validated using a *t*-test (*p* < 0.05, Benjamini-Hochberg corrected for FDR), after an analysis of variance (ANOVA) test, implemented in the Proteome Discoverer software (Thermo Scientific). The result lists were filtered and proteins were only kept for further investigation that fulfilled the following characteristics: identified by at least two peptides or by one peptide representing at least 10% protein coverage.

Functional annotation of proteins was performed using BlastKOALA and the Kyoto Encyclopedia of Genes and Genomes (KEGG) pathway database^60^. Proteome raw data has been deposited at MassIVE (https://massive.ucsd.edu/ProteoSAFe/static/massive.jsp?redirect=auth) under the dataset ID MSV000086166.

## Supplementary information

Figure S1

Figure S2

Figure S3

Figure S4

Table S1

Table S2

Table S3

Table S4

Table S5
