# Supplementary figures and images for: Tissue-specific signatures of metabolites and proteins in asparagus roots and exudates
Source: Hortic Res. 2021 Apr 1;8:86. doi: 10.1038/s41438-021-00510-5 (PMC8016990; doi:10.1038/s41438-021-00510-5)

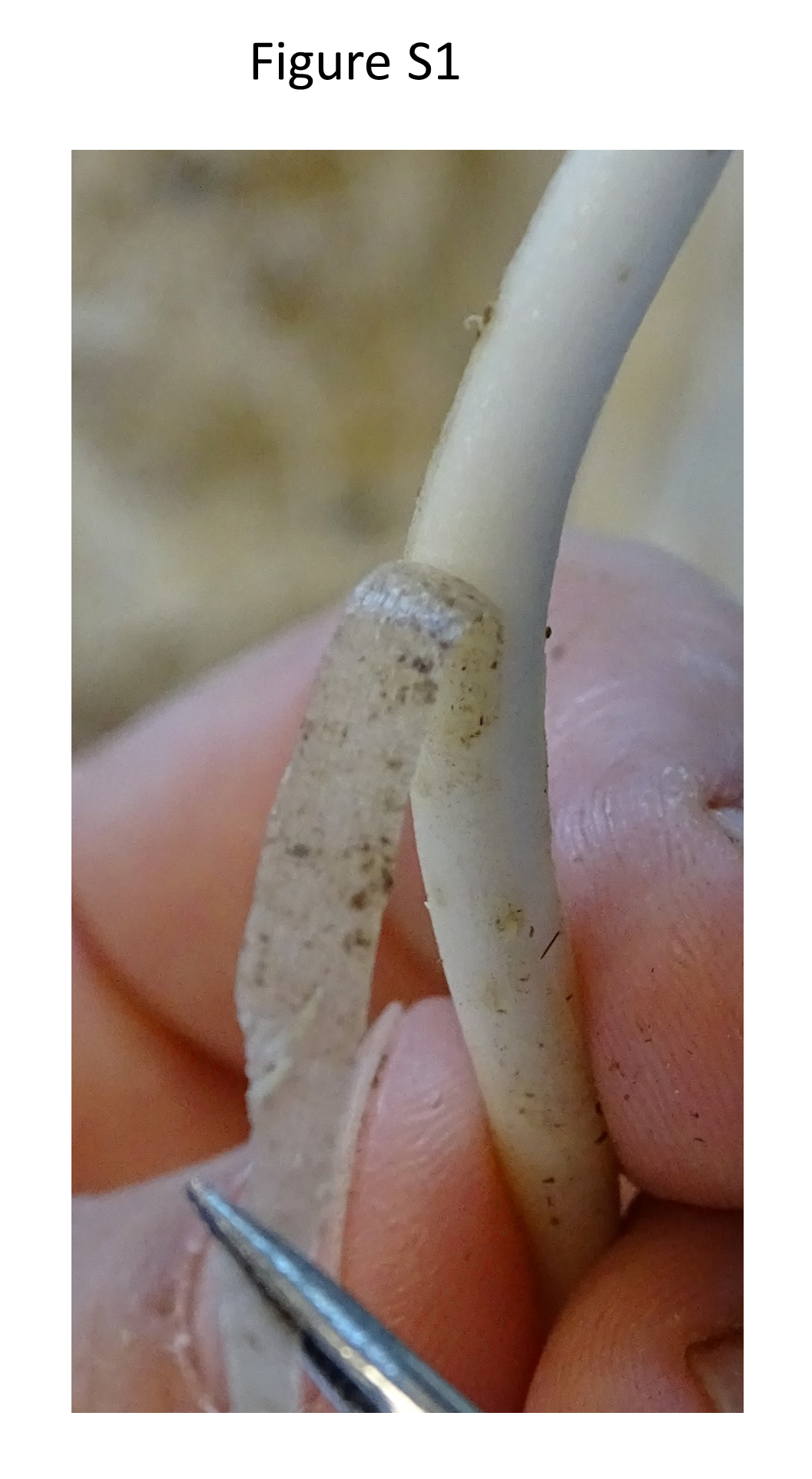

Supplement: Supplementary file 1 — Figure S1 [file 41438_2021_510_MOESM1_ESM.tif]

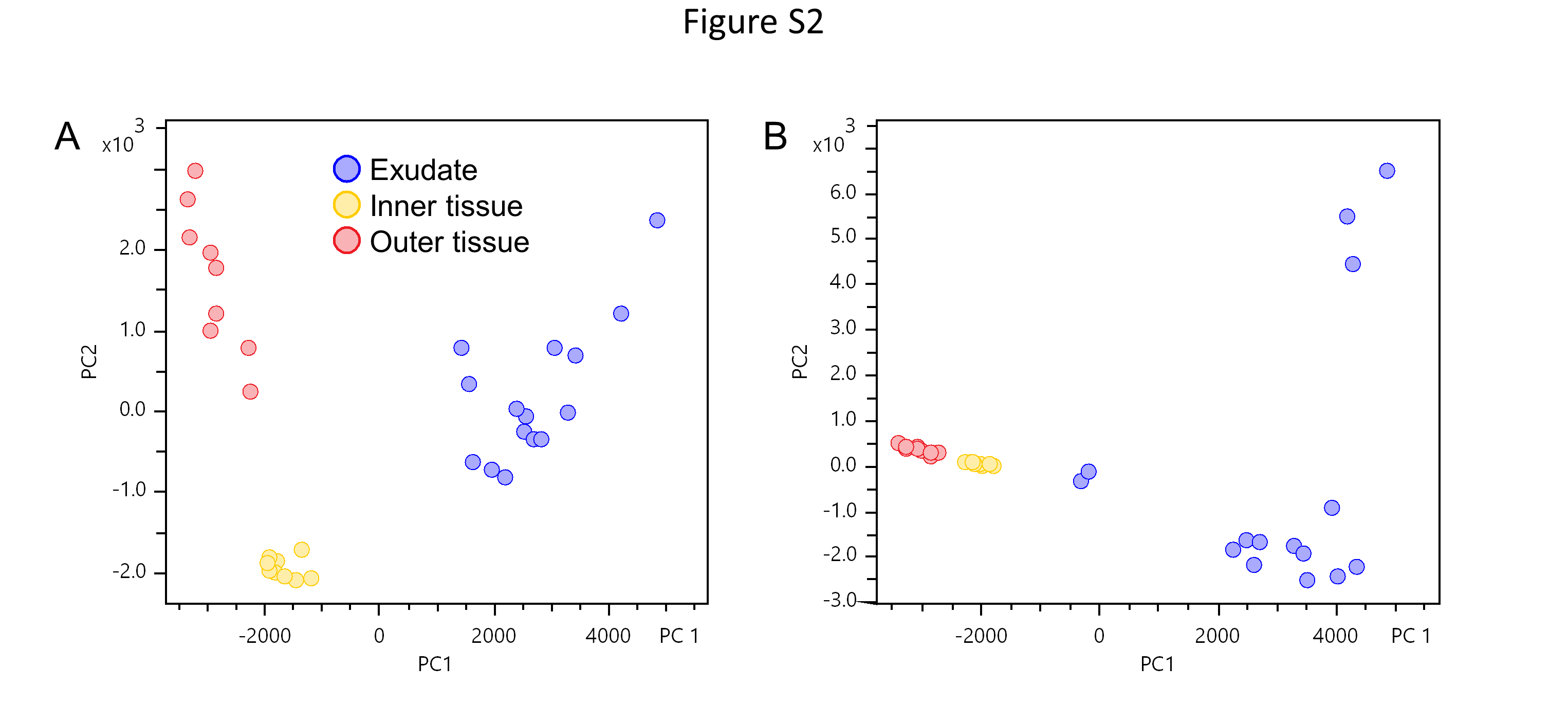

Supplement: Supplementary file 2 — Figure S2 [file 41438_2021_510_MOESM2_ESM.tif]

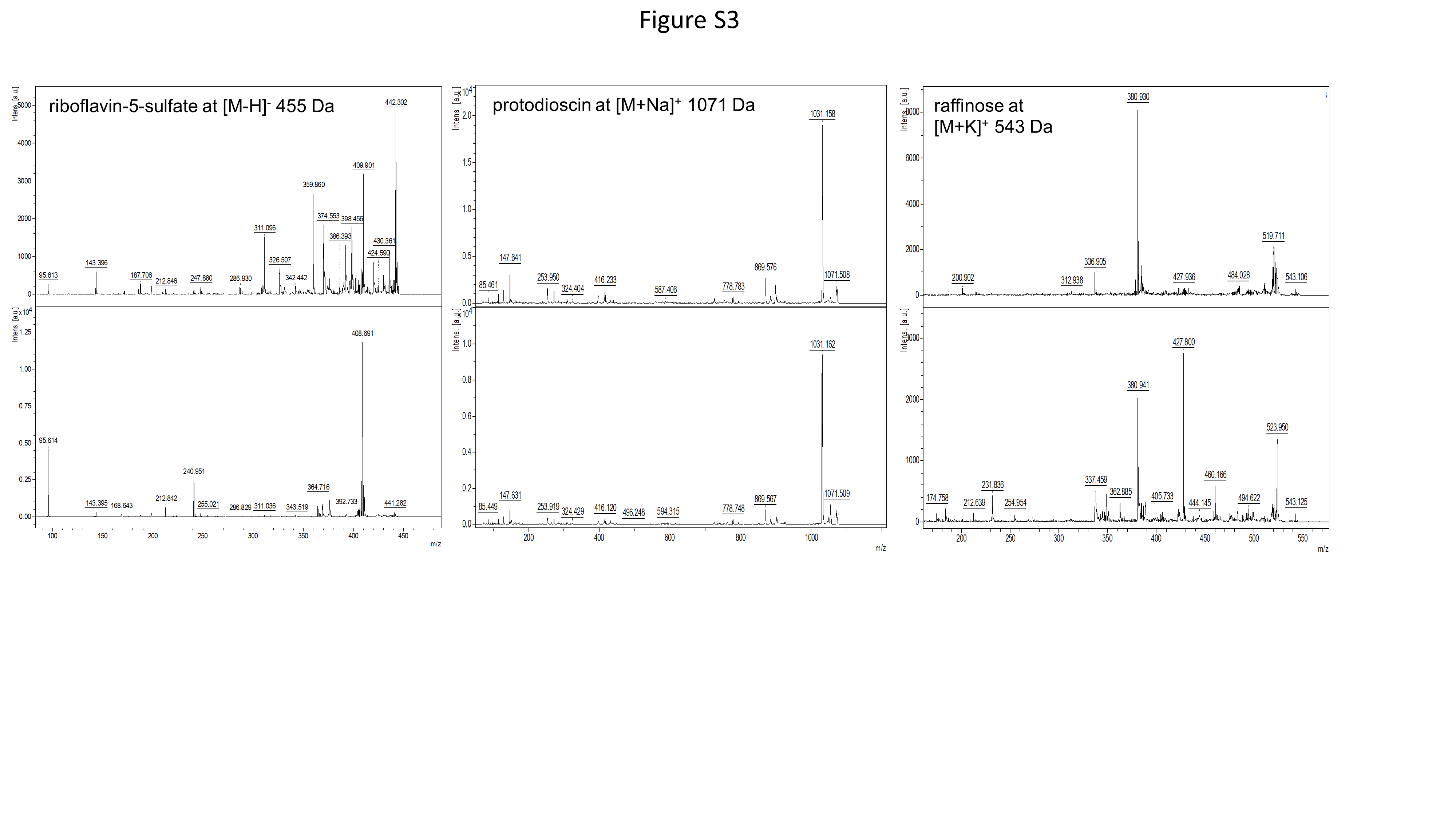

Supplement: Supplementary file 3 — Figure S3 [file 41438_2021_510_MOESM3_ESM.tif]

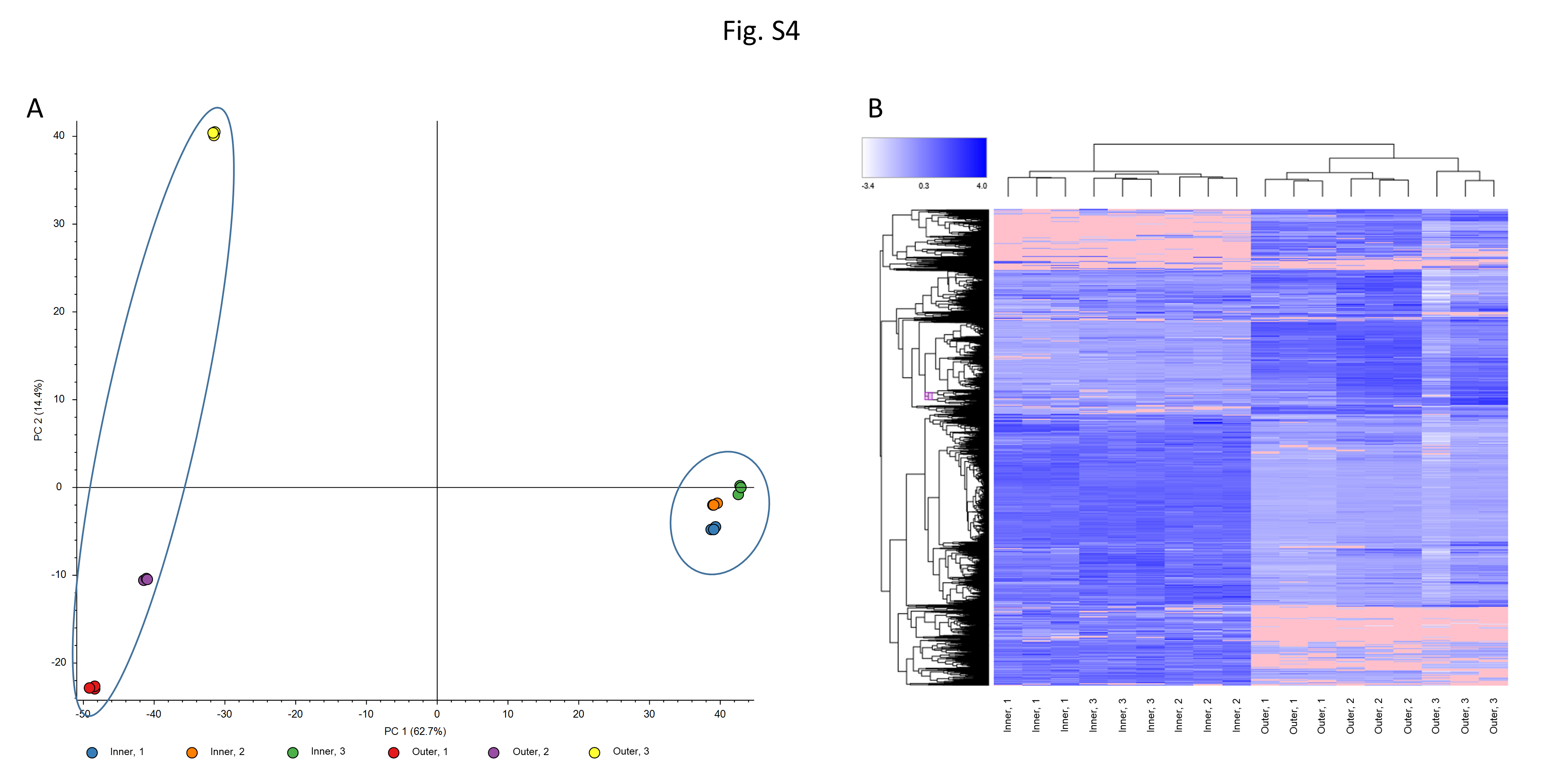

Supplement: Supplementary file 4 — Figure S4 [file 41438_2021_510_MOESM4_ESM.tif]
