# Supplementary material for: Tissue-specific signatures of metabolites and proteins in asparagus roots and exudates
Source: Hortic Res. 2021 Apr 1;8:86. doi: 10.1038/s41438-021-00510-5 (PMC8016990; doi:10.1038/s41438-021-00510-5)
Supplement: Supplementary file 9 — Table S5 [file 41438_2021_510_MOESM9_ESM.docx]

Table S5: Abundances of proteins with putative UDP-glycosyltransferase, cytochrome P450 or glutathione-S-transferase activity in outer and inner tissue of asparagus roots. Asterisks (*) indicate significant different protein expression between tissues (t-test; p<0.05).

| **Accession** | **Description** | **Putative Substrate** | **Abundance Inner Tissue** | **Abundance Outer Tissue** | **Abundance Ratio: Outer/Inner** |
| --- | --- | --- | --- | --- | --- |
| UDP-glycosyltransferase | |  |  |  |  |
| gi:1150750624 | UDP-glycosyltransferase 92A1 | unknown | - | 200 | - |
| gi:1150676881 | UDP-glucose:2-hydroxyflavanone C-glucosyltransferase | 2-hydroxyflavanones | 20.8 | 179.2 | 8.62* |
| gi:1150694989 | UDP-glycosyltransferase 71A15 | naringenin, naringenin chalcone, eriodictyol, eriodictyol chalcone, apigenin, luteolin, kaempferol, quercetin, isoliquiritigenin, butein and caffeic acid | 22 | 178 | 8.09* |
| gi:1150717891 | UDP-rhamnose: rhamnosyltransferase 1 | unknown | 22.7 | 177.3 | 7.81* |
| gi:1150720372 | UDP-glycosyltransferase 91A1 | unknown | 32 | 168 | 5.25* |
| gi: 1150692797 | crocetin glucosyltransferase | crocetin, 4-coumaric acid, caffeic acid, ferulic acid | 32.3 | 167.7 | 5.19 |
| gi:1150690491 | scopoletin glucosyltransferase | scopoletin, esculetin | 35.9 | 164.1 | 4.57* |
| gi:1150748877 | UDP-glucose:glycoprotein glucosyltransferase | unknown | 41.7 | 158.3 | 3.80 |
| gi:1150736900 | 7-deoxyloganetin glucosyltransferase | genipin and 7-deoxyloganetin | 51.5 | 148.5 | 2.88* |
| gi:1150677398 | scopoletin glucosyltransferase | scopoletin, esculetin | 89.5 | 110.5 | 1.23 |
| gi:1150673361 | scopoletin glucosyltransferase | scopoletin, esculetin | 109 | 91 | 0.84 |
| gi:1150693030 | UDP-glycosyltransferase 85A2 | quercetin-3-O, quercetin-7-O | 117.7 | 82.3 | 0.70 |
| gi:1150677396 | scopoletin glucosyltransferase | scopoletin, esculetin | 200 | - | - |
|  |  |  |  |  |  |
| Cytochrome P450 | |  |  |  |  |
| gi:1150681655 | cytochrome P450 71A1 | cyanogenic glycoside biosynthesis | - | 200 | - |
| gi:1150670766 | cytochrome P450 71A1 | tryptamine | 2.4 | 197.6 | 82.33 |
| gi:1150675556 | cytochrome P450 71A1 | cyanogenic glycoside biosynthesis | 4.3 | 195.7 | 45.51 |
| gi:1150714305 | cytochrome P450 84A1 | phenylpropanoids | 8.1 | 191.9 | 23.69* |
| gi:1150734984 | cytochrome P450 98A2 | p-coumaric esters of shikimic/quinic acids | 19.5 | 180.5 | 9.26* |
| gi:1150705833 | cytochrome P450 86B1 | long chain fatty acids | 20.8 | 179.2 | 8.62 |
| gi:1150674855 | cytochrome P450 71A1 | unknown | 38.7 | 161.3 | 4.17 |
| gi:1150748158 | cytochrome P450 | fatty acids | 64.1 | 135.9 | 2.12* |
| gi:1150726437 | cytochrome P450 709B1 | unknown | 83.6 | 116.4 | 1.39 |
| gi:1150689368 | cytochrome P450 72A15 | indole alkaloids | 102.1 | 97.9 | 0.96 |
| gi:1150694000 | cytochrome P450 90B2 | campestanol, 6-oxocampestanol brassinosteroids | 113.7 | 86.3 | 0.76 |
| gi:1150690507 | cytochrome P450 90B1 | campestanol, 6-oxocampestanol brassinosteroids | 174.8 | 25.2 | 0.14* |
|  |  |  |  |  |  |
| Glutathione-S-transferase | |  |  |  |  |
| gi:1150745665 | glutathione S-transferase GST 23 | unknown | - | 200 | - |
| gi:1150689602 | glutathione S-transferase GST 23 | unknown | - | 200 | - |
| gi:1150729275 | glutathione S-transferase U8 | unknown | - | 200 | - |
| gi:1150721989 | glutathione S-transferase | unknown | - | 200 | - |
| gi:1150740734 | glutathione S-transferase U17 | unknown | 3.2 | 196.8 | 61.50* |
| gi:1150700069 | glutathione S-transferase | unknown | 8.6 | 191.4 | 22.26* |
| gi:1150689598 | glutathione S-transferase GST 23-like isoform X1 | unknown | 12.3 | 187.7 | 15.26 |
| gi:1150668770 | glutathione S-transferase 3 | unknown | 12.6 | 187.4 | 14.87 |
| gi:1150683576 | glutathione S-transferase zeta class-like isoform X1 | unknown | 22.8 | 177.2 | 7.77* |
| gi:1150699556 | glutathione S-transferase T1 | 1-chloro-2,4-dinitrobenzene, p-nitrobenzyl chloride, cumene hydroperoxide, linoleic acid-13-hydroperoxide | 39.8 | 160.2 | 4.03* |
| gi:1150718160 | glutathione S-transferase parA | unknown | 41.5 | 158.5 | 3.82 |
| gi:1150668763 | glutathione S-transferase 3 | unknown | 96.9 | 103.1 | 1.06 |
| gi:1150727243 | glutathione S-transferase DHAR2 | dehydroascorbate | 136.5 | 63.5 | 0.47 |
| gi:1150682857 | glutathione S-transferase DHAR2 | dehydroascorbate | 184.8 | 15.2 | 0.08 |
| gi:1150678135 | glutathione S-transferase F10 | unknown | 185.3 | 14.7 | 0.08 |
| gi:1150748888 | glutathione S-transferase F10 | unknown | 194.9 | 5.1 | 0.03 |
